# INCREASED HIPPOCAMPAL ATROPHY IN HIGH INTENSITY INTERVAL TRAINING GROUP 4 YEARS AFTER 5-YEAR EXERCISE INTERVENTION

**DOI:** 10.1093/geroni/igad104.3429

**Published:** 2023-12-21

**Authors:** Asta Håberg, Line Reitlo, Jasmine Pani, Dorthe Stenvold

**Affiliations:** Norwegian University of Science and Technology, Trondheim, Sor-Trondelag, Norway; Norwegian University of Science and Technology, Trondheim, Sor-Trondelag, Norway; Norwegian University of Science and Technology, Trondheim, Sor-Trondelag, Norway; Norwegian University of Science and Technology, Trondheim, Sor-Trondelag, Norway

## Abstract

The Generation 100 Study is a randomized controlled trial (NCT01666340) investigating the effects of a 5-year exercise intervention on several health outcomes, including brain volumes from MRI. Participants (born 1936-42) were living independently at inclusion. They were randomized (1-1-2) to supervised exercising with either high intensity interval (HIIT) or moderate continuous training (MICT) twice a week, or to a control group adhering to the national guidelines of ≥30 min of moderate to vigorous physical activity at least 5 days a week. The intervention lasted for 5 years, during which participants underwent physical and clinical examinations at baseline, 1-, 3-, 5-years. 105 participants (n: HIIT=33, MICT=24, Cont=48) also underwent brain MRI at 3T. Linear mixed models were used and the interaction between group and time on brain volumes was the main outcome. We have previously reported that the HIIT group had significantly greater hippocampal atrophy rate compared to the control group during the intervention. Participants were invited back 4 years after end of intervention and underwent the same examinations including brain MRI . A total 73 participants (n: HIIT24=, MICT=19, Cont=30) took part. The HIIT group trained at a higher intensity than the other groups. There were no associations between objective VO2 or any self-reported exercise measurements and hippocampal volume/atrophy rate at any timepoint. Still, the hippocampal atrophy rate remained greater in the HIIT group than in the controls 4 years after end of the exercise intervention.

